# Letter to the editor: More data on vaccine efficacy/effectiveness of COVID-19 vaccines against asymptomatic SARS-CoV-2 infection

**DOI:** 10.2807/1560-7917.ES.2021.26.35.2100826

**Published:** 2021-09-02

**Authors:** Ying-Wen Chen, Wen-Chien Ko

**Affiliations:** 1 Department of Internal Medicine, National Cheng Kung University Hospital, College of Medicine, National Cheng Kung University, Tainan, Taiwan; 2 Department of Medicine, College of Medicine, National Cheng Kung University, Tainan, Taiwan

**Keywords:** COVID-19 vaccines, vaccine efficacy, vaccine effectiveness, asymptomatic SARS-CoV-2 infection

**To the editor**:****We read with interest the article by Harder et al. who reported on the interim results (from 1 January to 14 May 2021) of their living systematic review on coronavirus disease (COVID-19) vaccine efficacy/effectiveness (VE) in preventing severe acute respiratory syndrome coronavirus 2 (SARS-CoV-2) infections [1]. Notably, data on VE against asymptomatic infections in seven of 30 studies were discussed. We argue that additional clinical data on asymptomatic infection could have been extracted from two of the included studies.

For VE against asymptomatic SARS-CoV-2 infection, there were no data on the Vaxzevria vaccine (ChAdOx1-S, AstraZeneca, Cambridge, United Kingdom (UK)) described in Table 2. However, in the randomised controlled trial by Emary et al. as listed in Table 1, the VE of Vaxzevria vaccine at 2 weeks or more after the second dose against asymptomatic or unknown infection in the UK was 28.9% (95% confidence interval (CI): −77.1–71.4) for the SARS-CoV-2 Alpha variant (Phylogenetic Assignment of Named Global Outbreak (Pango) lineage designation B.1.1.7) and 69.7% (95% CI: 33.0–86.3) for other variants, mainly the B.1.177 variant [2]. One could argue the CI were wide and overlapping since three quarters of the patients had no sequencing data and those cases of infection with unknown symptoms may have had minor symptoms. Therefore, the significance of VE data was debatable.

In another study reported by Dagan et al., also included in Table 1, the VE of the Comirnaty vaccine (BioNTech, Mainz, Germany/Pfizer, Puurs, Belgium) against asymptomatic infection was estimated to be 29% (95% CI: 17–39) at 14–20 days after the first dose, 52% (95% CI: 41–60) at 21–27 days after the first dose, and 90% (95% CI: 83–94) at 1 week or later after the second dose [3]. This study included 1,193,236 participants, more than any other study listed in Table 2 and was the only matched case–control study available for the VE against asymptomatic infection.

In addition, in the sponsor briefing of the Spikevax (mRNA-1273, Moderna, Cambridge, United States (US)) vaccine presented to the US Food and Drug Administration (FDA) on 17 December 2020, the VE against asymptomatic infection was 63.3% (95% CI: 32.3–80.1) after the first dose in the vaccinated group (14/14,134, 0.099%) versus in the placebo group (38/14,073, 0.270%) [4]. This document is available on the FDA website. Since the assessment report on the Janssen vaccine (Janssen-Cilag International, Beerse, Belgium) by the European Medical Agency (EMA) was included in this systematic review, we believe that the FDA document for the Moderna vaccine would also be worth considering until formal analysis is available.

We appreciate the efforts by the authors to conduct this living systematic review. While the main SARS-CoV-2 variant of concern in the majority of studies during the report period was the Alpha variant, rather than the currently widespread Delta variant, continued efforts to collect the effectiveness data of current COVID-19 vaccines are welcome.
